# Long amplicon nanopore sequencing of *Botrytis cinerea* and other fungal species present in infected grapevine leaf samples

**DOI:** 10.1093/biomethods/bpad042

**Published:** 2024-01-05

**Authors:** Vladimer Baramidze, Luca Sella, Tamar Japaridze, Nino Abashidze, Daviti Lamazoshvili, Nino Dzotsenidze, Giorgi Tomashvili

**Affiliations:** Department of Plant Protection, , Agricultural University of Georgia, Kakha Bendukidze University Campus, Tbilisi 0159, Georgia; Department of Land, Environment, Agriculture and Forestry, University of Padua, Padova, Italy; Department of Plant Protection, , Agricultural University of Georgia, Kakha Bendukidze University Campus, Tbilisi 0159, Georgia; Department of Plant Protection, , Agricultural University of Georgia, Kakha Bendukidze University Campus, Tbilisi 0159, Georgia; Department of Plant Protection, , Agricultural University of Georgia, Kakha Bendukidze University Campus, Tbilisi 0159, Georgia; Department of Plant Protection, , Agricultural University of Georgia, Kakha Bendukidze University Campus, Tbilisi 0159, Georgia; Department of Virology and Molecular Biology, National Center for Disease Control and Public Health (NCDC), Tbilisi 0198, Georgia

**Keywords:** Botrytis cinerea, grapevine, nanopore sequencing, mycobiota

## Abstract

*Botrytis cinerea* is a well-known plant pathogen responsible for grey mould disease infecting more than 500 plant species. It is listed as the second most important plant pathogen scientifically and economically. Its impact is particularly severe in grapes since it affects both the yield of grape berries and the quality of wines. While various methods for detecting *B. cinerea* have been investigated, the application of Oxford Nanopore Technology (ONT) for complete ribosomal operon sequencing, which has proven effective in human and animal fungal research and diagnostics, has not yet been explored in grapevine (*Vitis vinifera*) disease research. In this study, we sequenced complete ribosomal operons (∼5.5 kb amplicons), which encompass the 18S, ITS1, 5.8S, ITS2, and 28S regions, from both pure cultures of *B. cinerea* and infected grapevine leaf samples. Minimap2, a sequence alignment tool integrated into the EPI2ME software, served as a taxonomy classifier, utilizing the custom reference database FRODO. The results demonstrate that *B. cinerea* was detectable when this pathogen was not the dominant fungal species in leaf samples. Additionally, the method facilitates host DNA-free sequencing and might have a good potential to distinguish other pathogenic and non-pathogenic fungal species hosted within grapevine’s infected leaves, such as *Alternaria alternata, Saccharomyces cerevisiae, Saccharomyces boulardii, Mucor racemosus,* and *Ascochyta rabie.* The sequences were uploaded to the NCBI database. Long amplicon sequencing method has the capacity to be broadened to other susceptible crops and pathogens, as a valuable tool for early grey rot detection and mycobiome research. Future large-scale studies are needed to overcome challenges, such as comprehensive reference databases for complete fungal ribosomal operons for grape mycobiome studies.

## Introduction

Diseases caused by pathogenic fungi pose a significant risk to grape production, leading to economic losses [1, 2]. *Botrytis cinerea* is the second most important plant pathogen responsible for causing grey mould rot in various economically significant crops [3]. In vineyards, this plant pathogen can infect grape leaves and, most frequently, flowers, resulting in latent infections that become aggressive during fruit ripening and berry maturation [4]. In addition to this primary pathogen, the populations present at the sites of infection, known as the microbiome, can significantly impact disease outcomes [5]. Also, the prevalence of yeasts and moulds in grapevine microbiome studies holds significant importance, given their potential roles in biocontrol and metabolic activities crucial to the winemaking process [6, 7].

Improvements in DNA sequencing techniques expanded *B. cinerea* detection and plant microbiome research, but a specific issue characterizes each. The Sanger sequencing method cannot process the microbiome data; sequencing of fungal genetic markers such as ITS1 and ITS2 requires a pure culture and cannot distinguish species in a mixture of amplicons, limiting its utility [8]. Massive parallel sequencing of short specific genetic markers, such as ITS1 and ITS2, has been generally used for research. While shorter amplicons offer high read quality and high throughput, this approach, commonly employed by Illumina and pyrosequencing techniques, often fails to assign taxonomy reliably at the species level [9].

In mycobiome research, single-molecule sequencers Nanopore and PacBio have their respective strengths and disadvantages in long amplicon sequencing [10]. Nanopore excels in real-time data acquisition and portability, making it ideal for fieldwork and rapid diagnostics. On the other hand, PacBio also stands out for exceptionally long reads and low error rates, making it a powerful tool for in-depth genomic studies of fungi. Both Nanopore and PacBio might require low DNA input and have high phasing capabilities [11, 12]; however, it is important to note that the PacBio instruments may not be as widely available as Nanopore due to high costs associated with it, thus limiting its access for researchers in certain regions [13].

Nanopore sequencing can be achieved either through whole metagenome sequencing, adaptive sampling, or amplicon-based sequencing. The whole-genome sequencing (WGS) protocol for fungal identification is robust. However, it generates an excess of sequenced data from the host organism genome, which does not allow optimal Nanopore flow cell utilization [14, 15]. An alternative to WGS is the enrichment approach provided by the ONT adaptive sampling concept, which is thought to represent a software-controlled enrichment. However, the software and library preparation steps are under investigation [16]. Long amplicon sequencing methods for fungal profiling using ONT involve sequencing the complete ribosomal operon (∼5500 bp), encompassing the 18S, ITS1, 5.8S, ITS2, and 28S regions [9]. The potential of long-read sequencing for fungal microbiome profiling was demonstrated on animal and human specimens [17, 18]. Investigating mycobiota on external otitis in dogs has proved that the method has the potential to characterize fungal communities in diverse samples, be it healthy or clinically affected [17]. The fungal communities were also characterized in human specimens (sputum) with ONT, revealing the capacity of long-read sequencing for the accurate identification of fungal diseases [18].

Consequently, the past years have seen improvements in pathogens detection from diseased plant tissues in microbial community studies. To date, there has been no research examining key grape diseases using this long amplicon ONT sequencing protocol. Our research aims to address key questions, including the detectability of *B. cinerea* with long amplicon sequencing, even if it coexists with other fungal species within grapevine leaf samples. Furthermore, this study explores the potential for identifying other fungal species present in infected grapevine leaves, as a ground for grey rot control and future microbiome studies.

## Material and methods

### Sample collection

*B. cinerea* B05.10 strain, used as control, was kindly provided by the University of Padova. In addition, two pure cultures of *B. cinerea* used as positive controls were kindly provided by Agricultural University of Georgia. These isolates were grown on Potato dextrose agar (PDA) at 21°C for 7 days, with a 12-h day photoperiod. An aseptic sampling of infected grapevine leaf tissues was performed on-site at small vineyards in different regions of Georgia: Kakheti and Imereti. A total of 10 asymptomatic grapevine leaves were collected at BBCH stage 7 (development of fruits), from plants exhibiting symptoms of *B. cinerea* infection in the previous year 2021. Before processing, the plant samples' surfaces were sterilized by treating them with 0.1% sodium hypochlorite for 30 s and rinsing them three times with sterile distilled water. Tissue fragments (200–400 mg) were aseptically removed from the samples using sterile scalpels or scissors, transferred to sterile plastic bags, pulverized with a hammer, and suspended in a pre-lysis buffer (OxGEn).

### DNA extraction and qPCR

DNA extraction from pre-lysed plant samples and from pure fungal cultures was performed using OxMag Pathogen DNA Purification Kit (OxGEn) following the manufacturer's instructions. The quality and quantity of the extracted DNAs were assessed using the NanoDrop ND-1000 instrument (NanoDrop Technologies) and Qubit 4 Fluorometer (Invitrogen by Thermo Fisher Scientific). Ten grapevine leaf samples were tested with *B. cinerea* TaqMan PCR Kit (Norgen Biotek Corp.) according to the manufacturer’s protocol. Two positive samples AW1 and AW2 (with *B. cinerea* detected at 34 and 36 Ct, respectively) were selected for downstream applications.

### Sanger sequencing library construction

The pure *B. cinerea* culture strains and the B05.10 control strain were sequenced with the Sanger sequencing method. Polymerase chain reaction (PCR) was performed using fungal-specific forward primer ITS1F (CTTGGTCATTTAGAGGAAGTAA) and reverse primer ITS4 (TCCTCCGCTTATTGATATGC), which amplified the ITS1 and ITS2 regions of nuclear ribosomal RNA genes [15]. The amplification reaction includes 30 ng of total DNA template, HOT FIREPOL^®^ DNA Polymerase (Solis Biodyne), 2.5 mM MgCl2 mM, and a 0.1 µM concentration of each primer. PCR was carried out in a GENE PRO TC-E-4l Thermal Cycler (BIOER) with an initial denaturation step at 95°C for 3 min, followed by 35 cycles of 95°C for 30 s, 58°C for 30 s, and 72°C for 1 min, and a final extension at 72°C for 10 min. Amplicons were submitted to Macrogen Europe BV (Nederland, Amsterdam) on ABI PRISM 3730XL Analyser (Applied Biosystems) with BigDye Terminator v3.1 Cycle Sequencing Kit (Applied Biosystems).

### Long-read amplicon sequencing library construction

Amplification and sequencing were performed for grapevine leaf fungal communities and *B. cinerea* pure cultures. The fungal-specific forward primer LR12 (GACTTAGAGGCGTTCAG) and the reverse primer SR1R (TACCTGGTTGATTCTGCCAGT) [19] were used at a concentration of 0.1 µM each, with up to ∼ 30 ng of total DNA template and OneTaq^®^ Hot Start DNA Polymerase (New England Biolabs). PCR was performed using a Thermal Cycler (BIOER), with an initial denaturation step at 94°C for 30 s, followed by 25 cycles of 94°C for 30 s, 50°C for 45 s, and 68°C for 5 min, and a final extension at 68°C for 5 min. The resulting amplicons were visualized on a 1% agarose gel by electrophoresis and analysed by GelRed staining.

Sequencing of the amplicons was performed by ONT sequencing with a MinION device (Oxford Nanopore Technologies) using a Ligation Sequencing Kit (LSK-109, Oxford Nanopore Technologies) for preparation of the amplicon library. The DNA was processed for end repair and dA-tailing using the NEBNext End Repair/dA-tailing Module (New England Biolabs). A purification step using 1X Agencourt OxMag XP beads (OxGEn) was performed. Native Barcoding kit (SQK-NBD114.24, Oxford Nanopore Technologies) was used for sample barcoding. For adapter ligation, Blunt/TA ligase master mix (New England Biolabs) was used. The 48-h sequencing protocol was run using MinKNOW software version 23.11.2.

### Bioinformatics and taxonomic-based analysis

The Albacore v.2.2.1 software was used for base-calling and de-multiplexing fast5 files. Porechop version 0.2.4 was employed for barcode and adapter trimming. Minimap2, integrated into the EPI2ME software, served as a taxonomy classifier, utilizing a custom reference database and the assignment taxonomy tool. The mapping parameters used for taxonomic classification in EPI2ME are integrated into the software and aligned against a reference that has been uploaded using the FASTA Reference Upload analysis with minimap2 version 2.12. The default parameters embedded in the EPI2ME software are utilized for this analysis.

The reference database, known as FRODO (Fungal rRNA Operon Database for ONT-sequences), was assembled by collecting 9072 fungal genome sequences from various sources, including NCBI, JGI, FungiDB, Ensembl Fungi, and the Broad Institute, to extract complete rRNA operon sequences [10]. All read classifications underwent a filtering process, and only those with coverage exceeding 70% and an identity of at least 90% were retained. Geneious Prime (version 2022 1.1) software was employed to analyse Sanger sequencing results. Consensus sequences were then subjected to NCBI BLAST search (Megablast—fast, high similarity matches) to determine the species of the samples. Pairwise alignments between the Sanger sequencing results and ONT were performed using BLAST software.

## Results

Prior to ONT sequencing, the control and pure cultures were confirmed by Sanger sequencing, while the presence of *B. cinerea* in the leaf samples was confirmed by a diagnostic commercial qPCR kit. Long-read sequencing of fungal amplicons resulted in the identification of 63 632 reads in pure culture samples and 65 173 in grapevine leaf samples (total number of reads from five samples: 130 805), with an average quality score of 11.4 and a read length of ∼5300 bp (base pairs). The distribution of *B. cinerea* in both pure cultures and grapevine leaf tissues is presented in Table 1. The AP1, AP2, and AP3 samples correspond to pure cultures, while AW1 and AW2 represent the total mycobiome of grapevine leaf samples. The average number of reads per sample was 25 761, providing sufficient sequencing depth to accurately assess the abundance of *B. cinerea* within the complex fungal microbiota associated with field samples.

**Table 1.** Fungal distribution in pure culture (AP1, AP2, and AP3) and grapevine leaf tissue samples (AW1 and AW2)

*Botrytis cinerea* distribution in the sequenced samples (%)					
Species	Sample AP1	Sample AP2	Sample AP3	Sample AW1	Sample AW2
*Botrytis cinerea*	97.77	98.05	97.44	5.52	4.79
Other Fungi^a^	1.89	1.04	0.74	88.98	90.78
Unknown^b^	0.34	0.91	1.82	5.50	5.08

a Fungal species not including *B. cinerea*.

b Unidentified fungal species.

The results indicate that *B. cinerea* was correctly assigned in the pure culture samples, with percentages ranging from 97.44% to 98.05%. Comparative analysis of Sanger and Nanopore in pure cultures revealed a high level of identity between Nanopore and Sanger sequences, with an average of 98.17% identity within 28% query coverage due to sequence read length difference. The high identity percentage is attributed to the sequencing depth of ONT consensus, despite the quality score of 11.4. In the grapevine leaf tissue samples, *B. cinerea* exhibited lower abundance (Ct values 34 and 36), accounting for only 5.52% to 4.79% of the sequenced reads. Other fungi, different from *B. cinerea*, were more prominent in the grapevine tissue samples, constituting 88.98% to 90.78% of the sequenced reads. 5.50% and 5.08% could not be assigned to known fungal species, indicating the presence of unidentified fungi in the analysed samples.

**Figure 1.**
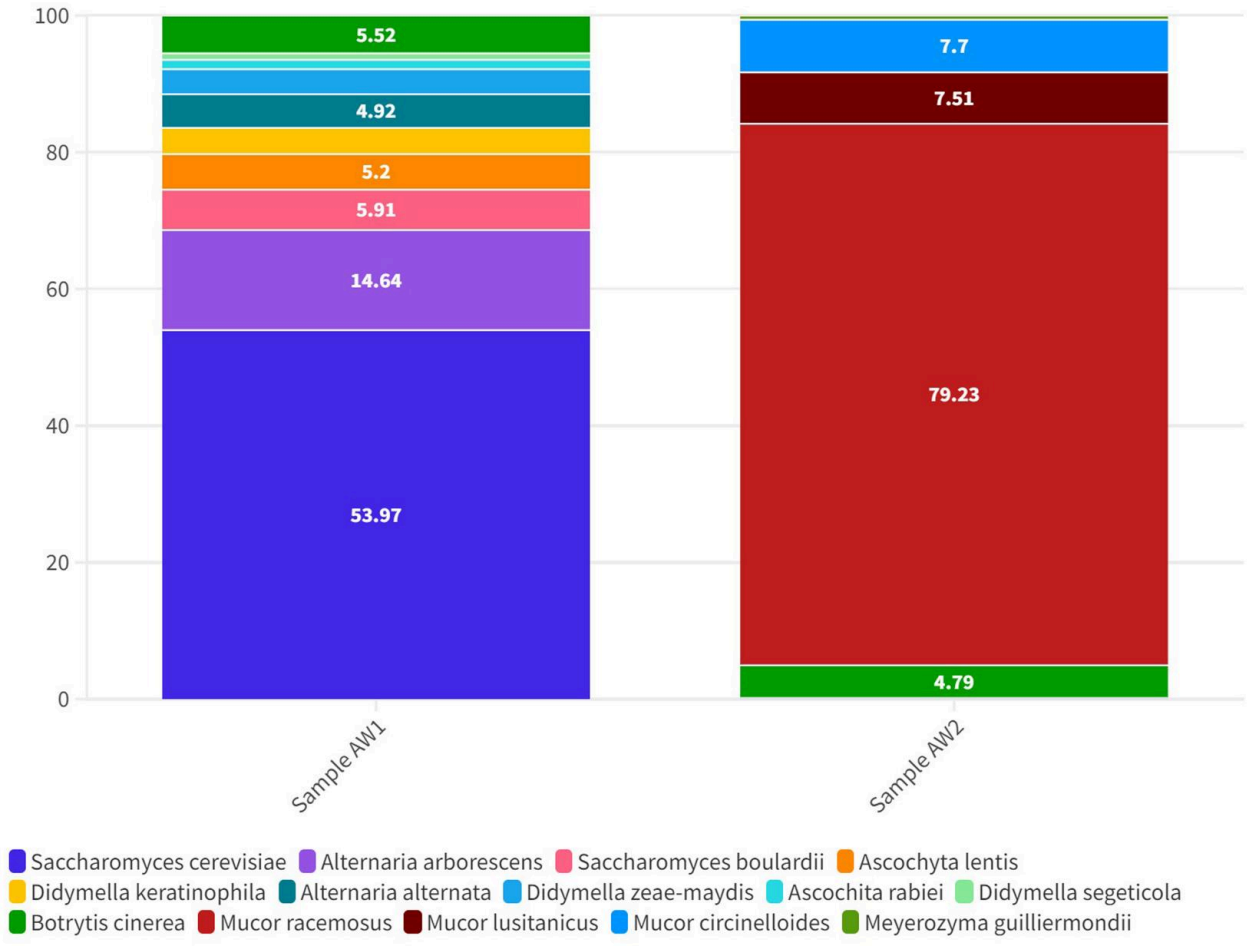
Abundance at the fungal species level in grapevine leaf samples using the complete ribosomal operon gene sequence as classification. Represented taxa occurred at >0.5% abundance in AW1 and AW2 samples.

The analysis of fungal species and their distribution within the studied samples provides valuable insights into the composition of the fungal mycobiota (Fig. 1). Among the identified fungal groups, the *Ascomycota* yeast *S. cerevisiae* was consistently present in the AW1 sample, with an abundance of 53,97%. In comparison, the Zygomycota *Mucor racemosus* was the dominant species in the AW2 sample (79.23%).

We detected a total of 52 different fungal species from the leaf samples, but only 14 species’ abundance was higher than 0.5%. Species with lower abundance than 0.5% were filtered to enhance the reproducibility of the reported information (Supplementary Table 1). *B. cinerea* was detected in the grapevine leaf samples at a relatively lower abundance than *S. cerevisiae* or *M. racemosus* and other fungal species in both AW1 and W2 samples (Fig. 1).

The average length of the *Botrytis cinerea* complete ribosomal operon was determined to be 5385 bp. We contributed our sequenced data to the NCBI nucleotide collection (nr/rt) database (LC743580.1; LC749799.1; LC750323.1; LC754729.1; LC756294.2).

## Discussion

Our paper describes a long-read amplicon Nanopore sequencing approach enabling the sequencing of complete ribosomal operons for the correct identification of *B. cinerea* from control pure cultures. Experimental design with three known pure cultures allowed to ensure the accuracy and reliability of the sequencing process and the reproducibility of the results. As expected, in these samples most of the reads belonged to *B. cinerea*, but 1.04 to 1.89% of reads were misclassified as other fungal species. This misclassification may have been the result of SNPS introduced during PCR amplification, a high degree of sequence similarity between *B. cinerea* and other *Botrytis* species, or it could be due to problems with non-comprehensive databases or inherited sequencing errors of Nanopore [13, 20].

Furthermore, our Nanopore sequencing method provides the potential for the detection and species-level taxonomic resolution of *B. cinerea* within grapevine leaf samples. In our research, we obtained sequences of 52 distinct fungal species within leaf samples infected by *B. cinerea*. *Saccharomyces cerevisiae* and *M. racemosus* were the most abundant species in the two different samples. The remaining species (*Saccharomyces boulardii, Ascochyta lentis, Didymella keratinophila, Didymella zeae-maydis, Ascochyta rabiei, Didymella segeticola, Mucor lusitanicus,* and *Mucor circinelloides*) were detected with abundance above 0.5%. The differences in the mycobiome composition in the AW1 and AW2 samples could be explained by the regional differentiation and agricultural practices. Several studies have suggested that the observed differences could be explained by the dominance of different species in different parts of a country. For instance, a significant association of *Aspergillus* and *Penicillium* spp. with the Chardonnay grapevine cultivar was observed in Napa; *Bacteroides, Actinobacteria, Saccharomycetes,* and *Erysiphe necator* were abundant in the Central Coast; *Botryotinia fuckeliana* and *Proteobacteria* were the dominant microorganisms in Sonoma [6].

Comparative analysis of Sanger and ONT sequencing have shown the methods’ capacity to accurately identify *B. cinerea* at the species level. Our analysis of sequences derived from the full-length rRNA operon revealed identity matches exceeding 95% when compared to species-representative sequences available in the FRODO database. We detected *B. cinerea* in complex microbiological samples, even when its abundance in the grapevine leaf samples was relatively low compared to other fungal species (5% according to ONT sequencing data, as also confirmed by qPCR with Ct values of 34 and 36). In our research, qPCR Ct values served as a control for the presence of *B. cinerea* infection, but we could not assign the Ct values to a fungal load in the infected samples. Further field experiments are needed with mock communities to establish whether the method is providing a real abundance of species. To exclude false-positive results, the enrichment of databases with closely related species sequences within different genera is crucial [21].

Complete ribosomal operon-based sequencing method serves as an effective tool for researching the phylogenetic composition of fungal taxa, which is challenging to be classified using ITS sequences alone [17]. The utilization of longer rRNA gene sequences, with the availability of both small-subunit (SSU) and large-subunit (LSU) reads, can significantly enhance the taxonomic resolution of fungal taxa [18]. In our study, we employed PCR primer pairs, specifically LR12/SR1R, which offered a comprehensive amplicon encompassing all regions of the complete ribosomal operon. We detected the presence of *B. cinerea* in leaf samples but also obtained host-free DNA sequences. This is a significant improvement over previous studies in the field of plant microbiome research, including Nanopore adaptive sampling protocols, where the issue of host DNA is still a challenge [16].

At the molecular level, the internal transcribed spacer (ITS) region continues to maintain its role as the universal fungal barcode marker, with the UNITE fungal database for nuclear ribosomal ITS regions containing over 230 000 sequences [22]. Notably, there is also a growing demand for longer sequences encompassing not only the complete ITS region but also the small-subunit (SSU) and large-subunit (LSU) regions to provide enhanced taxonomic resolution and information. In our study, we relied on the FRODO database (Fungal rRNA Operon Database for ONT-sequences) as our reference resource, which encompasses 9072 fungal amplicon sequences [10]. While FRODO offers a more focused dataset that aligns well with ONT sequencing, it underscores the persisting need for enrichment of databases to ensure the accurate identification of fungal species. This requirement remains a crucial step for future improvements in fungal taxonomy and mycobiome research.

In conclusion, the development and optimization of the Nanopore long amplicon-based fungal identification approach show significant promise across a wide range of applications in plant disease diagnostics, particularly within the grape industry. In regions with prevalent grapevine diseases, this methodology can enable host DNA-free, real-time diagnosis, significantly reducing confirmation time and offering crucial insights for pathogen surveillance and mycobiome research.

## Supplementary Material

bpad042_Supplementary_Data
