# Correction to: Long amplicon nanopore sequencing of *Botrytis cinerea* and other fungal species present in infected grapevine leaf samples

**DOI:** 10.1093/biomethods/bpae010

**Published:** 2024-02-23

This is a correction to: Vladimer Baramidze, Luca Sella, Tamar Japaridze, Nino Abashidze, Daviti Lamazoshvili, Nino Dzotsenidze, Giorgi Tomashvili, Long amplicon nanopore sequencing of *Botrytis cinerea* and other fungal species present in infected grapevine leaf samples, *Biology Methods and Protocols*, Volume 9, Issue 1, 2024, https://doi.org/10.1093/biomethods/bpad042

In the originally published version of this manuscript, a statement of funding was erroneously omitted. The **Acknowledgements** section should read: “We thank Dr Nana Bitsadze for providing us pure cultures of fungal strains, confirmed by morphology. The research was funded by the Shota Rustaveli National Science Foundation of Georgia under the “Young Scientist Research Grant # YS 18-1446” agreement.” instead of: “We thank Dr Nana Bitsadze for providing us pure cultures of fungal strains, confirmed by morphology.”

This has been emended in the article.

