# Supplementary material for: Long amplicon nanopore sequencing of Botrytis cinerea and other fungal species present in infected grapevine leaf samples
Source: Biol Methods Protoc. 2024 Jan 5;9(1):bpad042. doi: 10.1093/biomethods/bpad042 (PMC10789308; doi:10.1093/biomethods/bpad042)
Supplement: bpad042_Supplementary_Data [file bpad042_supplementary_data.docx]

**Supplementary Table 1.** Presence and the abundance of known fungal species in the grapevine leaf tissue, classified using the complete ribosomal operon gene sequence. The indicated taxa are those with an abundance exceeding 0.5% in at least one of the two samples.

| Species distribution in the sequenced samples (%) | | |
| --- | --- | --- |
| Species | Sample AW1 | Sample AW2 |
| *Saccharomyces cerevisiae* | 53,97 | 0,10 |
| *Alternaria arborescens* | 14,64 | 0,00 |
| *Saccharomyces boulardii* | 5,91 | 0,00 |
| *Ascochyta lentis* | 5,20 | 0,00 |
| *Didymella keratinophila* | 4,67 | 0,00 |
| *Alternaria alternata* | 4,92 | 0,02 |
| *Didymella zeae-maydis* | 3,62 | 0,00 |
| *Ascochita rabiei* | 0,88 | 0,08 |
| *Didymella segeticola* | 0,67 | 0,00 |
| *Botrytis cinerea* | 5,52 | 4,79 |
| *Mucor racemosus* | 0,00 | 79,23 |
| *Mucor lusitanicus* | 0,00 | 7,51 |
| *Mucor circinelloides* | 0,00 | 7,70 |
| *Meyerozyma guilliermondii* | 0,00 | 0,57 |
